# Machine learning potential for modelling H_2_ adsorption/diffusion in MOFs with open metal sites†

**DOI:** 10.1039/d3sc05612k

**Published:** 2024-03-05

**Authors:** Shanping Liu, Romain Dupuis, Dong Fan, Salma Benzaria, Mickaele Bonneau, Prashant Bhatt, Mohamed Eddaoudi, Guillaume Maurin

**Affiliations:** a UMR 5253, CNRS, ENSCM, Institute Charles Gerhardt Montpellier, University of Montpellier Montpellier 34293 France guillaume.maurin1@umontpellier.fr; b LMGC, Univ. Montpellier, CNRS Montpellier France; c Division of Physical Science and Engineering, Advanced Membrane and Porous Materials Center, King Abdullah, University of Science and Technology (KAUST) Thuwal 23955-6900 Kingdom of Saudi Arabia mohamed.eddaoudi@kaust.edu.sa

## Abstract

Metal–organic frameworks (MOFs) incorporating open metal sites (OMS) have been identified as promising sorbents for many societally relevant-adsorption applications including CO_2_ capture, natural gas purification and H_2_ storage. This has been ascribed to strong specific interactions between OMS and the guest molecules that enable the MOF to achieve an effective capture even under low gas pressure conditions. In particular, the presence of OMS in MOFs was demonstrated to substantially boost the H_2_ binding energy for achieving high adsorbed hydrogen densities and large usable hydrogen capacities. So far, there is a critical bottleneck to computationally attain a full understanding of the thermodynamics and dynamics of H_2_ in this sub-class of MOFs since the generic classical force fields (FFs) are known to fail to accurately describe the interactions between OMS and any guest molecules, in particular H_2_. This clearly hampers the computational-assisted identification of MOFs containing OMS for a target adsorption-related application since the standard high-throughput screening approach based on these generic FFs is not applicable. Therefore, there is a need to derive novel FFs to achieve accurate and effective evaluation of MOFs for H_2_ adsorption. On this path, as a proof-of-concept, the soc-MOF-1d containing OMS, previously envisaged as a potential platform for H_2_ adsorption, was selected as a benchmark material and a machine learning potential (MLP) was derived for the Al-soc-MOF-1d from a dataset initially generated by *ab initio* molecular dynamics (AIMD) simulations. This MLP was further implemented in MD simulations to explore the H_2_ binding modes as well as the temperature dependence distribution of H_2_ in the MOF pores from 10 K to 80 K. MLP-Grand Canonical Monte Carlo (GCMC) simulations were then performed to predict the H_2_ sorption isotherm of Al-soc-MOF-1d at 77 K that was further confirmed using sorption data we collected on this sample. As a further step, MLP-based molecular dynamics (MD) simulations were conducted to anticipate the kinetics of H_2_ in this MOF. This work delivers the first MLP able to describe accurately the interactions between the challenging H_2_ guest molecule and MOFs containing OMS. This innovative strategy applied to one of the most complex molecules owing to its highly polarizable nature, paves the way towards a more systematic accurate and efficient *in silico* assessment of MOFs containing OMS for H_2_ adsorption and beyond to the low-pressure capture of diverse molecules.

## Abstract 2

Machine learning potential is required to accurately describe the interactions between the challenging H_2_ molecules and metal organic frameworks containing open metal sites.

## Graphical abstract

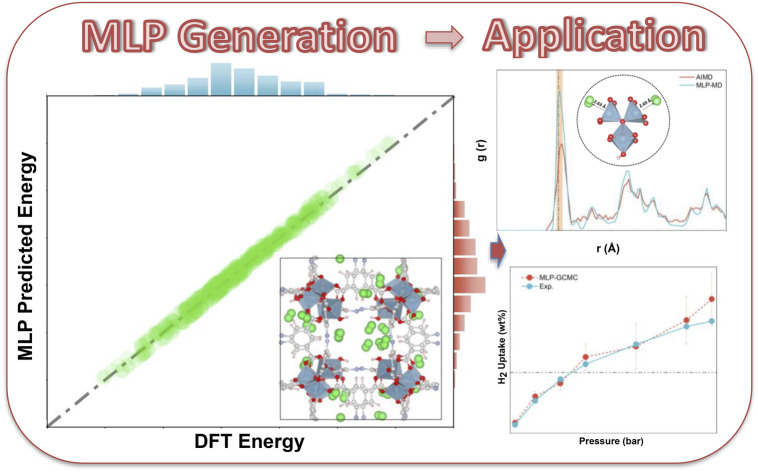

## Introduction

Metal–organic frameworks (MOFs) constructed from metal ions/metal clusters connected to organic linkers have been widely studied and continue to gain momentum as a class of porous frameworks.^1–5^ Their unique tunability in terms of chemical functionality, architecture and pore size/shape makes them potentially applicable in many fields, including gas capture/storage, separation in gas and liquid phases, catalysis, biomedicine and sensing among others.^6–8^ One sub-class of MOFs contains open metal sites (OMS) also called coordinatively unsaturated sites (CUS) in the cluster nodes, to which guest molecules can readily bind.^9–11^ The formation of this strong metal–molecule bond has been demonstrated to play a key role not only in initiating a myriad of catalytic reactions in the MOF pores but also in selectively adsorbing a desired molecule.^9^ Typically, Mg(ii)_2_(dobpdc) with its pore wall decorated with Mg-OMS is one of the prototypical MOFs for the selective capture of CO_2_ over a range of other molecules (N_2_, CH_4_, H_2_O…),^12^ while MIL-100 (Cr iii) owing to its Cr-OMS was demonstrated to be highly selective for N_2_ over CH_4_ of great interest for natural gas purification.^13^ MOFs incorporating OMS have also shown promise for the storage/delivery of hydrogen (H_2_),^14^ a highly relevant energy vector, especially as a replacement for traditional fossil fuels. This topic is of high importance since net-zero hydrogen with a greenhouse gas (GHG) footprint of zero is expected to provide up to 24% of the total EU energy demand.^15^ Typically, H_2_ storage currently involves the use of high pressure and/or cryogenic temperatures that implies high additional costs and safety issues.^16^ The challenge in this field is to identify porous materials able to adsorb reversibly high H_2_ uptake and concurrently maximize deliverable H_2_ capacity. Since the first coordinatively saturated MOF tested for H_2_ adsorption,^17^*i.e.*, MOF-5, the MOF community designed a series of porous materials incorporating OMS over the last two decades with the objective of substantially enhancing H_2_ binding energy/adsorption enthalpy for achieving high adsorbed hydrogen densities.^9,14^ Typically, V_2_Cl_2.8_(btdd) containing a high concentration of V(ii) sites demonstrated high usable hydrogen capacities that exceed that of compressed storage under the same operating conditions.^18^

This list of examples highlights the key role played by OMS in many adsorption-related properties of MOFs and the need to gain an in-depth understanding of the OMS–guest molecule interactions towards the refinement of MOFs with improved performances.^9–11^ Molecular simulation has proven to be a complementary tool to sophisticated characterization techniques to precisely characterize the interactions between guest molecules and MOFs containing OMS. To effectively simulate these rather complex host/guest systems, a reliable force field is required to accurately describe the potential energy surface (PES). Here, the challenge lies in a correct description of the weaker intermolecular forces between the guest molecules and the host framework, *e.g.*, coulombic and van der Waals interactions as compared to the strong intramolecular forces within the host framework, *e.g.*, metal ion–linker bonding. The quantum *ab initio* approach, such as the dispersion-corrected density functional theory (DFT) enables a precise determination of such interaction forces, however, its applicability is restricted to small systems containing less than hundreds of atoms due to its prohibitive computational cost.^19–21^ Additionally, *ab initio* molecular dynamics (AIMD) simulations are also limited to pico-second time-scales.^22^ These shortcomings make the *ab initio* approach very time consuming to explore the guest adsorption in MOFs at long-time and large-length scales. It is indeed extremely challenging to model MOF properties at length and time scales comparable with experimental observations as discussed by Van Speybroeck *et al.*^23^ However, classical force field (FF) Monte Carlo (MC) and Molecular Dynamics (MD) simulations offer a good compromise that have been widely employed to explore the adsorption and diffusion of guest molecules in MOFs at micro-second time scales and several nanometer length scales.^24–28^ The large majority of these reported theoretical studies rely on the application of Lorentz Berthelot mixing rules between generic FFs, *e.g.*, universal force field (UFF) and Dreiding among others for the MOF framework^29,30^ and diverse FF models for the guests to describe the host/guest interactions. Although this simplified approach has been shown to describe quite well the interactions between small guest molecules and coordinatively saturated MOFs, it cannot anymore be applicable to MOFs containing OMS that induce high polarization in the adsorbed molecules.^31^ This statement hampers the computational-assisted identification of MOFs containing OMS for a target application since the standard high-throughput screening approach based on these generic FFs is not applicable. Indeed such MOF-OMS/guest molecule interaction requires a specific FF parameterization that is far from being a trivial task.^32^ Therefore, there is a critical need to move beyond classical approaches and derive FFs combining high efficiency and high accuracy, capable of describing the overall interactions that are in play in MOF-OMS/guest molecule systems. One promising approach to achieve this objective is the development of machine learning potentials (MLPs), which are trained on database preliminary generated by DFT calculations. Development of MLPs for describing PES in condensed matter was pioneered by Behler and Parrinello.^33^ This was achieved by considering physically meaningful descriptors which represent very well the atomic structure. By directly fitting the relationships between the structure and energy, the MLP generally enables complex interactions to be reproduced more accurately than classical force fields and more efficiently (less expensive computational cost) than DFT. The MLP applied to MOFs is therefore expected to gain an unprecedented description of the MOF-OMS/guest molecule systems with high-accuracy accounting for the overall forces present in this complex system. So far, the development of MLPs for MOFs has been limited to only a very few cases.^34–39^ Behler *et al.*, first derived a high-dimensional neural network MLP to effectively describe the crystal structure of MOF-5.^34^ Fan *et al.* equally developed a MLP to explore the mechanical properties of a novel 2D MOF.^40^ Johnson *et al.* further combined MLP for the UiO-66 framework with classical FFs for rare gases to explore the host/guest interactions.^35^ Very recently, Vandenhaute *et al.* built a neural network MLP with parallelized sampling and on-the-fly training to explore the phase transformation for different MOFs.^36^ Zheng *et al.* implemented a novel MLP to explore the CO_2_ binding mode and diffusion in MOF-74 containing Mg(ii)-OMS.^37^ Similarly, Shaidu *et al.* developed a neural network MLP for the exploration of CO_2_ binding in amine-appended Mg_2_(dobpdc).^38^ Goeminne *et al.* also trained a MLP to accurately capture the adsorption behavior of CO_2_ in both ZIF-8 and Mg-MOF-74.^41^ These preliminary studies highlight that the MLP offers a great opportunity to explore the most complex MOF host–guest interactions at large scale.

Achieving an accurate description of the interactions between H_2_ and any host frameworks at the force field level is even more challenging since quantum-mechanical effects become significant for this smallest molecule particularly at cryogenic temperature and its polarizability in confined space plays also a key role.^42–44^ Many force fields are available in the literature for describing H_2,_ including the most sophisticated ones that implement the Feynman–Hibbs variational approach to take into account the quantum effects.^45,46^ However once combined with generic force fields to describe coordinatively saturated MOF frameworks, they frequently failed to reproduce the thermodynamics/dynamics of H_2_@MOF systems that motivated tedious force field re-parametrization based on experimental data for some specific systems.^47,48^ The complexity becomes even higher when one deals with the interactions between H_2_ and MOF-OMS that have been solely treated so far at the pure quantum-level.^49–52^

**Fig. 1:**
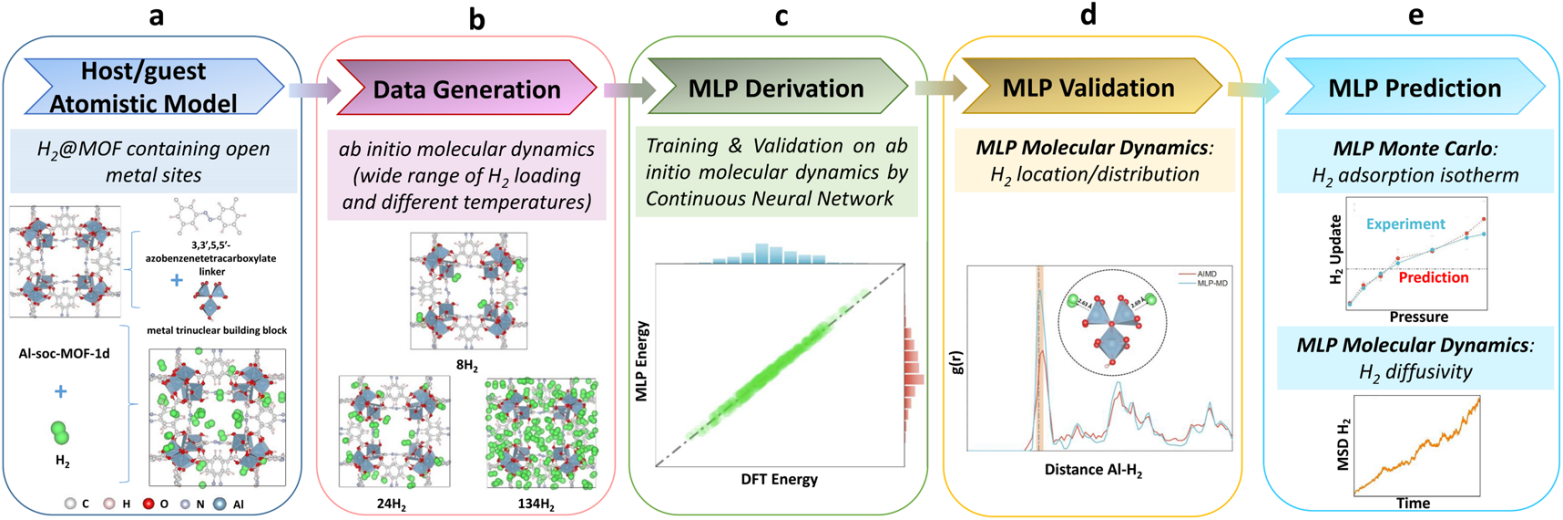
Workflow devised for the derivation and validation of a machine learning potential for H_2_@Al-soc-MOF-1d towards prediction. (a) Construction of the atomistic model for the host/guest system. (b) Generation of the training datasets using *ab initio* molecular dynamics simulations. (c) Training and validation of the MLP using a continuous neural network. (d) External validation of the trained MLP with its implementation in molecular dynamics simulations and (e) MLP-molecular dynamics and MLP-Monte Carlo predictions.

Herein, we aim to develop a MLP using a deep neural network to accurately explore the adsorption and diffusion behaviors of H_2_ in a prototypical MOF containing OMS. As a proof-of-concept, the Al-soc-MOF-1d platform composed of a 6-connected metal trinuclear molecular building block and a 4-connected rectangular-planar organic ligand, 3,3′,5,5′-azobenzenetetracarboxylate linker (shown in Fig. 1a) was selected. This MOF is seen as a benchmark material owing to the presence of open metal sites in the Al oxo-trimer nodes and the relatively moderate size of its unit cell (432 atoms and 10 178 Å^3^ cell volume) while this MOF platform is also attractive from an application standpoint since its In- and Fe-versions were previously demonstrated effective for H_2_ adsorption.^53,54^ Molecular dynamics simulations implementing MLPs initially trained from a series of configurations generated by AIMD simulations at different temperatures and different H_2_ loading enabled accurate capture of the binding modes of H_2_ towards the OMS and beyond to elucidate the H_2_ distribution in the overall porosity of the Al-soc-MOF-1d in a wide temperature range spanning from 10 K to 80 K.

MLP-MD simulations further delivered a microscopic picture of the diffusion of H_2_ in Al-soc-MOF-1d. Finally, MLP-Grand Canonical Monte Carlo (GCMC) simulations further predicted the H_2_ adsorption isotherm at 77 K that was validated by a good agreement with the adsorption isotherm freshly collected on a well-activated Al-soc-MOF-1d sample. This computational work delivers the first MLP able to accurately describe the interactions between H_2_ and MOF-OMS, a key to gaining an in-depth understanding of the MOF-OMS/H_2_ interactions of importance to further develop advanced MOF sorbents for efficient H_2_ storage.

## Results and discussion

### Overall computational strategy

Fig. 1 illustrates the overall workflow we implemented to train and validate a MLP to accurately describe the host–guest interactions in the prototypical H_2_@Al-soc-MOF-1d system. The first step (Fig. 1a) involved the construction of a reliable atomistic guest-loaded model. The unit cell considered for the cage-like Al-soc MOF,^14^ contains metal Al oxo-trimer nodes with 1 metal bounded to the OH counter-ions herein typically considered as representative counter-ions and 2 OMS. This simulation cell was loaded with different H_2_ loadings ranging from 8 to 134 molecules per unit cell, *i.e.* from 0.31 wt% to 5.25 wt%, that cover the overall adsorption regime of the analogue In-soc-MOF previously assessed by adsorption measurements.^55–57^ To achieve sufficient structural sampling for further MLP training, four initial H_2_@Al-soc-MOF-1d configurations were generated for each explored guest loading using random insertion, followed by DFT structure optimization and AIMD simulations (see the Computational methods section for details). The construction of the MLP then involved three steps: generation of high-quality quantum training data, creation of effective descriptors by fingerprinting the local atomic environment, and establishment of a robust mapping between the descriptors and atomic energies and forces. The training set on the H_2_@Al-soc-MOF-1d models was thus generated (Fig. 1b) by DFT optimization and AIMD simulations performed at different temperatures with a time step of 0.5 fs, accumulating 21k + data points to assemble a relatively large collection of configurations, energies, and forces (see the dataset in Computational method section).

The DeepMD-kit program was employed to build the descriptors based on the produced training data.^58,59^ Within DeepMD-kit, a continuous neural network was utilized to establish a robust mapping between the effective descriptors and atomic energies to generate the MLP (Fig. 1c). Concurrently, the accuracy of the fitted MLP was validated using the separate validation dataset (500 data points), assessing the performance and reliability of the MLP. The next step consisted of implementing the derived MLP in MD simulations to externally validate its reliability to describe the preferential location/distribution of H_2_ in the Al-soc-MOF-1d (Fig. 1d). Finally, the robustness of this MLP was challenged *via* its integration into a Monte Carlo (MC) scheme to predict the H_2_ adsorption isotherm of Al-soc-MOF-1d in the low-pressure region and up to 0.4 bar that was further compared to the corresponding newly collected experimental data. Complementary MLP-MD simulations further enabled H_2_ transport to be anticipated (Fig. 1e) in this MOF.

### MLP derivation using a neural network algorithm

**Fig. 2:**
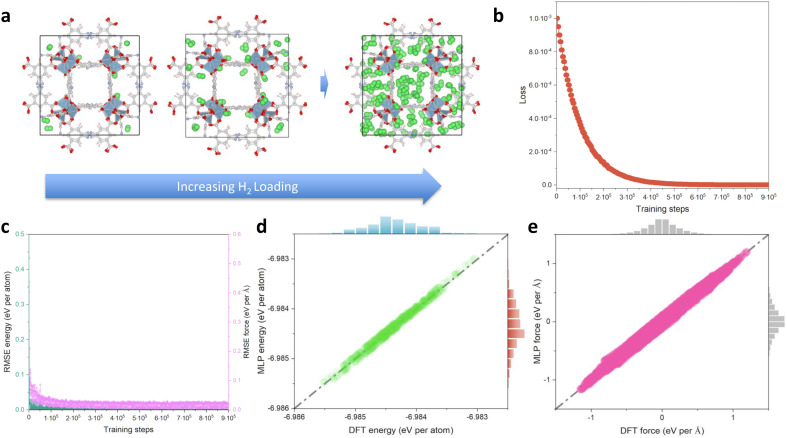
MLP derivation for H_2_@Al-soc-MOF-1d. (a) Illustration of the H_2_@Al-soc-MOF-1d host–guest model systems with different H_2_ loading (from 8 to 134 molecules per unit cell) selected for the generation of the training/validation data points by AIMD simulations. (b) Variation of the loss function over the training loops. (c) Training RMSE of energy and force changes over the training loops. (d and e) Training test with randomly 500 selected configurations from the training datasets (detailed information can be found in the Computational methods section).

Fig. 2a shows representative H_2_ loaded Al-soc-MOF-1d structures obtained by AIMD for the different considered gas uptakes. These structures are used in the subsequent dataset preparation. Fig. 2b shows the loss function over the training steps with an associated RMSE of energy lower than 0.10 meV per atom (Fig. 2c). To assess the accuracy of the so-trained MLP, 500 configurations were selected from the training dataset resulting in an associated RMSE of energy (force) of 0.08 meV per atom (0.02 eV Å^−1^) (Fig. 2d and e). We further randomly chose 500 configurations from the validation dataset also leading to a very low RMSE of 0.04 meV per atom. Notably, all these RMSE values for energy closely approach the convergence energy criteria generally applied in quantum calculations.^60^ As shown in Fig. 2d, e, S1 and S2,† the linear correlation between the MLP-predicted energies/forces and AIMD-calculated values is excellent, with R-squared values exceeding 0.99 for both training and validation tests. Therefore, the MLP demonstrates its ability to accurately predict energies across the entire energy range of the training and validation dataset, achieving satisfactory accuracy when compared to quantum calculations.

### MLP validation throughout MD simulations

**Fig. 3:**
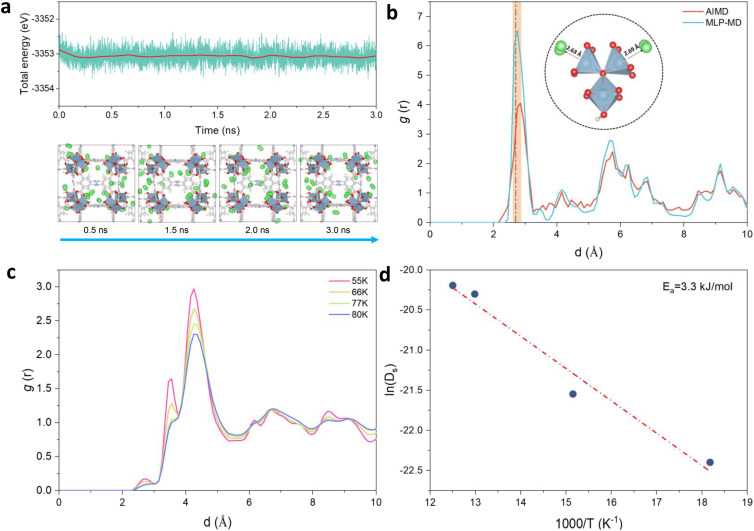
MLP-MD validation/prediction for H_2_@Al-soc-MOF-1d. (a) Energy fluctuation (up) and corresponding representative snapshots (bottom) of the MLP-MD for 24H_2_@Al-soc-MOF-1d at 10 K. H_2_ molecules are depicted in green spheres, the color code for the rest of the MOF atoms is the same as in Fig. 2. (b) Radial distribution functions (RDFs) calculated for the Al-OMS/H_2_ pair by MLP-MD (light-blue) and AIMD (red) both performed at 10 K. The dashed line, located at approximately 2.7 Å, corresponds to the equilibrium Al–H_2_ distance obtained in the DFT-geometry optimized structure (0 K). The inset delivers an illustration of these preferential interactions between H_2_ and the Al-OMS site. (c) RDFs calculated for the Al-OMS/H_2_ pair by MLP-MD simulations conducted at various temperatures (55, 66, 77, and 80 K, respectively). (d) Arrhenius plot of the self-diffusion for H_2_ simulated by MLP-MD simulations (see Fig. S5† for the associated MLP-MD simulated mean-squared displacements (MSD) plotted as a function of time).

MD simulations implementing the derived MLP were further performed to explore the average distribution of H_2_ in the pores of Al-soc-MOF-1d. Fig. 3a reports the energy fluctuation of the 24H_2_@ Al-soc-MOF-1d system over a 3 ns-long NVT MLP-MD simulation conducted first at very low temperature (10 K). Illustrative snapshots (Fig. 3a, bottom) selected over the MLP-MD trajectory show that H_2_ molecules remain mostly located next to the MOF pore wall, privileging interactions with the inorganic nodes (Fig. 3b). Analysis of the radial distribution function (RDF) calculated for the Al-OMS/H_2_ pair revealed a preferential interaction between the guest molecule and the Al-OMS with an average separating distance of ∼2.7 Å that aligns with that derived from the AIMD simulations conducted at the same temperature. Interestingly, this geometric adsorption feature is in excellent agreement with the conclusions drawn from the DFT-geometry optimized H_2_@Al-soc-MOF-1d structure at 0 K, associated with Al-OMS/H_2_ separating distance of 2.7 Å and H_2_ binding energy of −7.1 kJ mol^−1^. Notably, the overall MLP-MD and AIMD-derived RDF profiles for the Al-OMS/H_2_ pair match well (Fig. 3b). Indeed apart from the reproduction of the first sharp and intense RDF peak, MLP-MD simulations enable the low intensity peaks to be captured at 3.5 and 4.2 Å corresponding to the interactions between H_2_ and the two other Al atoms present in the same oxo-centered trimer as well as additional peaks within the 5 to 7 Å range assigned to the interactions between H_2_ and the Al atoms present in the neighboring oxo-centered trimers. The contribution in the range of 8.5–9.5 Å is associated with the distance between H_2_ and the most distant oxo-centered trimer in the periodic unit cell. This overall observation demonstrates unambiguously that the trained MLP successfully captures the quantum-derived structuring of H_2_ in the MOF pores as shown by an excellent reproduction of all RDF peak positions, widths and heights for the Al-OMS/H_2_ pair. Complementary MLP-MD simulations were performed to examine the transferability of MLP to a wider temperature range from 10 K to 80 K. Examinations of the energy evolution of the MD runs over the MD trajectories show that the integrity of the MOF framework is maintained and the Al-soc-MOF-1d and H_2_ interactions are well described within the overall temperature range (Fig. S3†). Analysis of the RDF plotted for the Al-OMS/H_2_ pair at different temperatures (Fig. 3c) shows that the intensity of the first main peak decreases as the temperature increases accompanied by a tiny shift towards longer separating distance. This observation is in line with a faster mobility of H_2_ within the MOF pores as temperature increases. This highlights that at lower temperature H_2_ molecules are much more localized next to the Al-OMS, which are the primary adsorption sites of this MOF. At higher temperature H_2_ molecules become more mobile and tend to distribute at longer distance to the Al-OMS. This behavior is in line with a relatively moderate binding energy (−7.1 kJ mol^−1^) that prevents to maintain a strong coordination of H_2_ towards the OMS once the thermal vibration increases. Furthermore, static analysis of the adsorption positions of H_2_ molecules validates the reliability of our MLP in accurately describing the H_2_ adsorption properties at various temperatures. This confirms the MLP's ability to capture the temperature-dependent behavior of H_2_ within the MOF system.

This structural analysis highlights that the derived MLP achieves an accurate description of the overall H_2_@Al-soc-MOF-1d interactions in a wide temperature range since it enables the temperature-dependent location of the guest to be finely captured in this MOF. Beyond this observation, the derivation of such a robust MLP paves the way towards the exploration of such complex host/guest systems at a longer timer scale (ns *vs.* ps scale) and lower computational cost (>1000 times faster) compared to AIMD simulations (*cf.* Fig. S4†). This is of key importance particularly for probing the guest diffusion in MOFs that operates at longer lengths and time scales than that accessible by AIMD simulations.

### MLP molecular dynamics/Monte Carlo predictions

MLP-MD simulations were thus performed to explore the dynamics of H_2_ in Al-soc-MOF-1d at different temperatures. Fig. S5† shows that the resulting mean squared displacement (MSD) follows a linear time-dependence, signature of a Fickian diffusion regime. The self-diffusion coefficient (*D*_s_) for H_2_ was thus calculated for all temperatures using the Einstein-relation. Herein *D*_s_ of H_2_ at 77 K is 1.5 × 10^−9^ m^2^ s^−1^ which is lower than the value previously reported for MOFs free of OMS sites, like MIL-53, MIL-47, and IRMOF, with *D*_s_ ranging from 5 × 10^−9^ to 5 × 10^−8^ m^2^ s^−1^ at 77 K as evaluated by both force field molecular dynamics simulations,^47,61,62^ and quasi-elastic neutron scattering experiments.^47^Fig. 3d reports the Arrhenius plot for *D*_s_ associated with an activation energy of 3.3 kJ mol^−1^. This energetic value is higher than that simulated for the MOFs free of OMS sites *i.e.*, MIL-53 (1.25 kJ mol^−1^),^47^ MIL-47 (0.68 kJ mol^−1^),^47^ IRMOF-1 (2.55 kJ mol^−1^),^61^ IRMOF-8 (2.10 kJ mol^−1^),^61^ and IRMOF-18 (3.09 kJ mol^−1^)^61^ Interestingly It falls within the same range of values reported previously for zeolite NaX (4.0 kJ mol^−1^) by experiments where H_2_ can also interact with the extra-framework cation Na^+^.^63^ Both slower self-diffusion coefficient and higher activation energy predicted for Al-soc-MOF-1d compared to other MOFs mentioned above are in line with the specific interactions between H_2_ and Al-OMS that tend to retain more H_2_ localized around the inorganic node and indeed slow down its diffusivity.

**Fig. 4:**
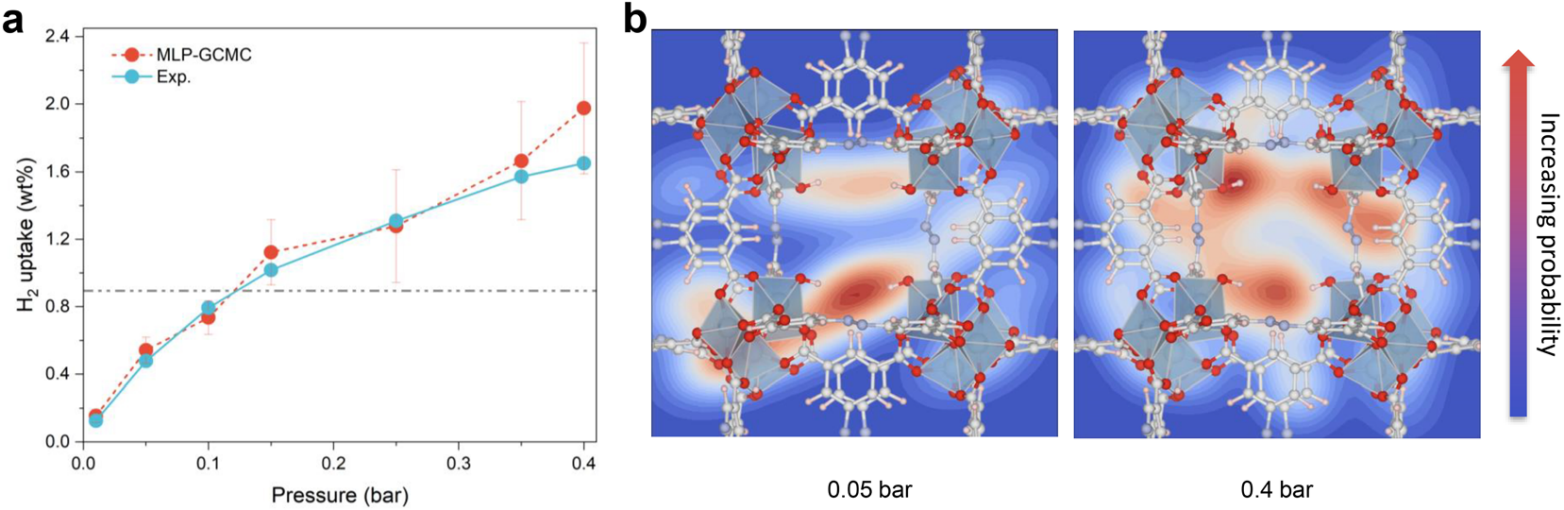
MLP-GCMC prediction. (a) Simulated H_2_ adsorption isotherms for Al-soc-MOF-1d (red symbols) *vs.* experimental volumetric measurements (blue symbols) at 77 K, ranging from 0.01 to 0.4 bar. (b) Simulated probability distribution of H_2_ at 0.05 (left) and 0.4 bar (right), respectively.

Finally, the MLP was implemented in a Grand-Canonical Monte Carlo (GCMC) scheme to predict the adsorption isotherm of H_2_ in Al-soc-MOF-1d at 77 K. As stated above, this is a great challenge for the MOF community working in the field of adsorption to accurately predict the adsorption isotherm at very low pressure for a MOF containing OMS.^31^ Herein the MLP-GCMC simulations were performed at 77 K for pressure ranging from 0.01 bar to 0.4 bar. The resulting MLP-GCMC simulated adsorption isotherm is reported in Fig. 4a. To gain more insights into the adsorption mechanism in play, we also analyzed the probability distribution of H_2_ at 0.05 bar and 0.4 bar, as shown in Fig. 4b. In these snapshots, the red and blue regions are associated with high and low probability to find H_2_ respectively. At very low pressure (0.05 bar), these plots confirm that H_2_ molecules preferentially adsorb in the vicinity of the Al-OMS. At higher pressure (0.4 bar), more H_2_ molecules are adsorbed, and these additional molecules occupy less energetically favorable adsorption sites in the pores especially in the center of the cages resulting in a more homogeneous adsorption pattern. To validate these GCMC simulations, the Al-soc-MOF-1d sample was synthesized and fully activated prior to collecting its H_2_ adsorption isotherm (see the ESI for details Fig. S6†). The very good agreement obtained between the MLP-GCMC calculated and the experimental H_2_ adsorption isotherms delivers a strong confirmation that the derived MLP accurately describes the interactions between H_2_ and Al-OMS that dominate the initial stage of adsorption. This conclusion is also supported by the simulated adsorption enthalpy at low coverage of −7.5 kJ mol^−1^ which is in line with the experimental value reported previously for the In-soc-MOF (−6.5 kJ mol^−1^)^56^ as well as the DFT-derived binding energy (−7.1 kJ mol^−1^).

## Conclusions

In summary, we derived a MLP first trained from a set of trajectories generated by *ab initio* molecular dynamics simulations to accurately describe the interaction between H_2_ and Al-soc-MOF-1d containing Al-OMS. A preliminary validation in terms of H_2_ binding mode and temperature dependent H_2_ distribution, was achieved by means of MLP-MD simulations performed at temperature ranging from 10 K to 80 K. MLP-MD and MLP-GCMC simulations were further conducted at 77 K to gain an unprecedented microscopic insight into the thermodynamics adsorption and dynamics of H_2_ in this MOF. This high-accuracy molecular simulation approach was validated by a good agreement between the predicted MLP-GCMC H_2_ adsorption isotherm and the corresponding experimental data collected on a well-activated Al-soc-MOF-1d sample. Decisively, such a derived MLP overcomes the limitations of generic force fields that inaccurately the interactions between small molecules and MOFs with OMS that has hampered so far accurate prediction on MOFs with OMS for diverse adsorption-related applications. This first MLP related to H_2_@MOF-OMS is of utmost importance since H_2_ is known to be highly polarizable, this phenomenon being accurately described by quantum mechanics. Beyond gaining an in-depth understanding of the MOF-OMS/H_2_ interactions to further develop advanced MOFs for efficient H_2_ storage, this computational strategy is expected to be more systematically applied for predicting the adsorption properties of many existing MOFs with OMS. More specifically for H_2_, the future direction might consider the development of MLPs from path integral AIMD to consider nuclear quantum effects of this complex molecule.^41,64,65^

## Computational methods

### DFT calculations

A unit cell of Al-soc-MOF-1d with the lattice parameter *a* = *b* = *c* = 21.67 Å and *α* = *β* = *γ* = 90° was loaded with different H_2_ adsorption loading ranging from 0.31%wt to 5.25 wt%. All the training and validation data-sets were generated by AIMD using the Vienna *Ab initio* simulation package (VASP) with projector augmented wave (PAW) pseudopotential.^66,67^ The generalized gradient approximation (GGA) with Perdew–Burke–Ernzerhof (PBE) functionals was used to describe the exchange–correlation interaction of the electrons.^68^ To account for the non-local, long-range electron corrections, Grimme empirical dispersion corrections (DFT-D3 method) were adopted in all DFT calculations.^69^ This DFT-D method has been demonstrated to accurately describe the H_2_ adsorption in MOF-74 and its analogues {M_2_(DHFUMA) [M = Mg, Fe, Co, Ni, Zn]} containing OMS.^50–52^ The Kohn–Sham orbitals (wave functions) were expanded in a plane-wave basis set with a cut-off energy of 480 eV. The Brillouin zone was limited to the *Γ* point. For geometry optimization at 0 K, the overall H_2_@Al-soc-MOF-1d systems were fully relaxed until both the energy and force reached the convergence criteria of 10^−5^ eV and 0.01 eV Å^−1^, respectively. These geometry optimized configurations served as the initial stage for the AIMD simulations. All AIMD simulations were performed in the canonical ensemble (NVT) with the Nosé–Hoover thermostat to control the temperatures from 10 K to 100 K. A timestep of 0.5 fs was employed to integrate the equations of motion.^70^ The datasets used for test and validation are both 500 data points. The validation dataset is randomly selected from the training data while the test dataset comes from new AIMD simulations performed at 77 K (24H_2_ per unit-cell). Detailed information and corresponding data can be found in Table S1† and a separate dataset link containing all relevant data on the Zenodo website.

### MLP calculations

The DeepMD-kit package was employed to train MLPs from the configurations generated by quantum calculations (including DFT-optimization and AIMD simulations at different temperatures). The training process involves optimizing the parameters in both the filter and fitting NNs to minimize the dimension-less loss function:
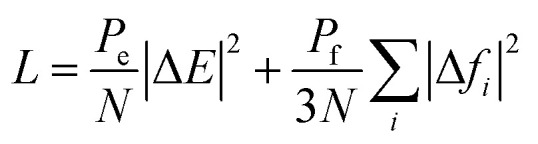
where |Δ*E*|^2^ and |Δ*f*_*i*_|^2^ represent the root mean square errors (RMSEs) of the energy and force, respectively. These errors quantify the discrepancies between the predicted values and the reference values obtained from the DFT calculations. *P*_e_ and *P*_f_ act as perfectors during the training process, which are functions of the dynamic loss function and continuously adapt during the optimization of the NNs.

In this work, the cutoff radius (*r*_cut_) for neighbor searching was set to 7.9 Å, and the smoothing starts at 3.1 Å (*r*_cut__smth). These values were set according to the pore size of Al-soc-MOF-1d. The maximum number of neighbor atoms (sel) within the radius cutoff was set to [70, 80, 80, 80, 90, 80] for different types [“Al”, “O”, “C”, “H”, “H_2_”, “N”] respectively. The size of the filter NNs (neuron) and fitting NNs (fitting_net: neuron) was chosen as {25, 50, 100} and {240, 240, 240}, respectively. To control the training process, the decay_rate and decay_steps were set to 0.95 and 5000 respectively. The initial values of perfectors P_e (start_pref_e) and P_f (start_pref_f) in the loss function were set to 0.02 and 1000 respectively. The learning rate (start_lr) starts at 0.001 and decreases exponentially over the training process. Further, the Adam stochastic gradient descent method was adopted for the training process.^58,71^

### MLP-MD simulations

The trained-MLP was implemented into a MD scheme with the MLP executed using the DeepMD-kit interface to the LAMMPS code.^72^ These MD simulations were carried out in the NVT thermostat ensemble using a timestep of 0.5 fs, and the duration of the MD simulations is at the ns-level.

### MLP-GCMC simulations

The trained-MLP was implemented in a MC scheme using an in-house developed MC code in conjunction with the LAMMPS program.^72^ For the GCMC simulations performed at 77 K, a series of random moves for H_2_ including insertion (30%), deletion (30%), translation (20%), and rotation (20%) were considered. The acceptance or rejection of these moves was determined by calculating the Boltzmann probability of different configurations. Additionally, the potential energy functions for different positions and orientations of gas molecules were considered to determine the probability density of gas molecules at various adsorption sites.

## Experimental methods

### MOF synthesis and characterization

A mixture of AlCl_3_·6H_2_O (13 mg, 0.054 mmol) and 3,30,5,50-azobenzenetetracarboxylic acid (10 mg, 0.028 mmol) was dissolved in *N*,*N*-dimethylformamide (DMF) (2 mL), acetonitrile (CH_3_CN) (2 mL), and acetic acid (1 mL). The solution was carefully transferred into Pyrex vial with phenolic cap lined with polytetrafluoroethylene (PTFE). The vial was then placed in a pre-heated oven at 150 °C for duration of 3 days, resulting in the formation of pure orange crystals. The as-synthesized crystals were washed 3 times with DMF (10 mL). Subsequently, the crystal solution was exchanged with acetonitrile (10 mL) for 6 days in a 65 °C oven. The final product, Al-soc-MOF-1d, was activated under vacuum after 240 °C activation for 12 h (Fig. S6†). Low pressure gas adsorption measurements for H_2_ were performed on a 3-Flex surface characterization analyzer (Micromeritics) at relative pressures up to 1 bar. The temperature bath for the H_2_ sorption was controlled using liquid nitrogen at 77 K.

## Data availability

Additional data can be found in the ESI.† The derived MLP potential and related data can be found at https://doi.org/10.5281/zenodo.10686480. This link includes the DFT calculation input files, all the AIMD simulation datasets, the MLP training input, the final trained MLP file, as well as the MLP-GCMC simulation scripts.

## Author contributions

M. E. and G. M. designed the research. S. L. carried out the DFT calculations, D. F. performed the MLP training and MLP-MD simulations. R. D. realized the MLP-GCMC simulations. S. B. synthesized the MOF sample, M. B. and P. B. performed the adsorption measurements. All authors participated in the writing of the manuscript. M. E. and G. M. supervised the research.

## Conflicts of interest

There are no conflicts to declare.

## Supplementary Material

SC-015-D3SC05612K-s001
