# Supplementary material for: Machine learning potential for modelling H2 adsorption/diffusion in MOFs with open metal sites
Source: Chem Sci. 2024 Mar 5;15(14):5294–302. doi: 10.1039/d3sc05612k (PMC10988610; doi:10.1039/d3sc05612k)
Supplement: SC-015-D3SC05612K-s001 [file SC-015-D3SC05612K-s001.pdf]

Electronic Supplementary Material (ESI) for Chemical Science.
This journal is © The Royal Society of Chemistry 2024

Supplementary Information

Machine Learning Potential for Modelling H_2 Adsorption/Diffusion in MOFs with Open Metal Sites

Shanping Liu,[a] Romain Dupuis,[a,b] Dong Fan,[a] Salma Benzaria,[c]
Mickaele Bonneau,[c] Prashant Bhatt,[c] Mohamed Eddaoudi,[c,*] and
Guillaume Maurin [a,*]

a. ICGM, Université de Montpellier, CNRS, ENSCM, Montpellier, 34293, France

b. LMGC, Univ. Montpellier, CNRS, Montpellier, France

c. Division of Physical Science and Engineering, Advanced Membrane and Porous Materials Center, King Abdullah, University of Science and Technology (KAUST), Thuwal 23955-6900, Kingdom of Saudi Arabia

Supplementary Table S1 | Data preparation for the MLP training of the H_2@Al-soc-MOF-1d system (including DFT-optimizations and AIMD runs at 10-100K).

H_2 loading [per unit-cell]	Number of atoms	Number of configurations
**8**	448	1575
**24**	480	11574
**40**	512	2236
**57**	546	2148
**103**	638	2204
**134**	700	1882

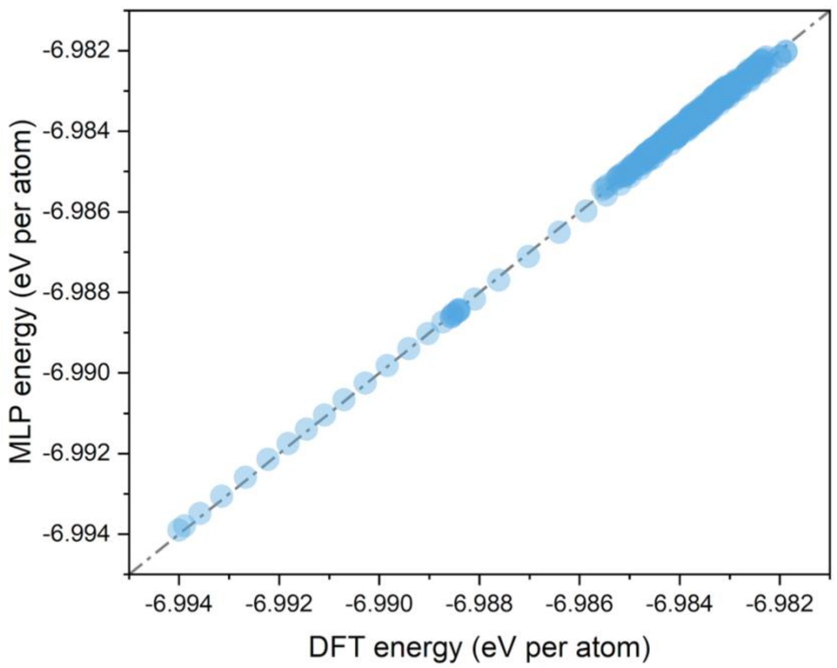

Supplementary Fig. S1 | MLP derived energies *vs* the corresponding DFT values in the validation dataset.

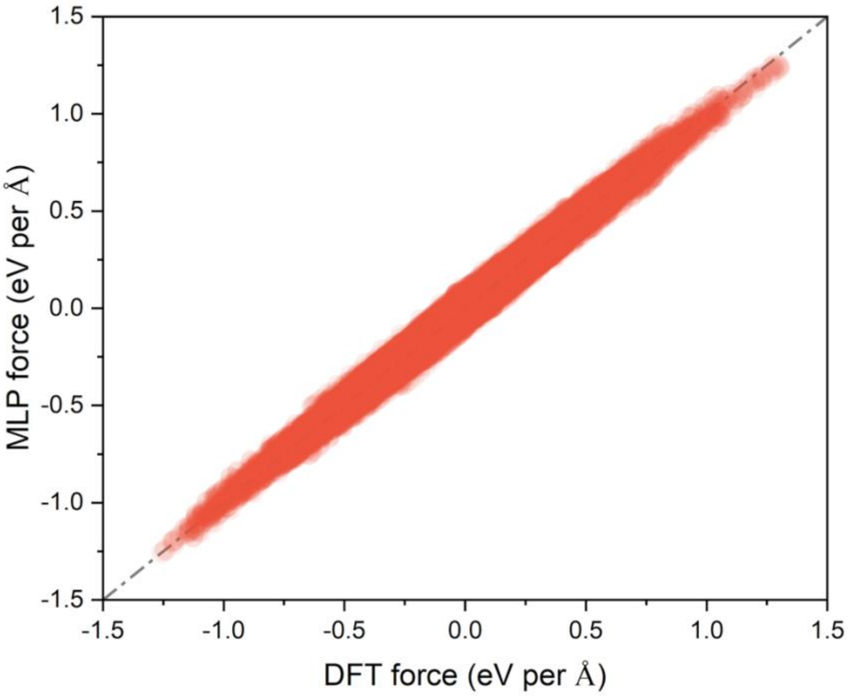

Supplementary Fig. S2 | MLP derived forces *vs* the corresponding DFT values in the validation dataset.

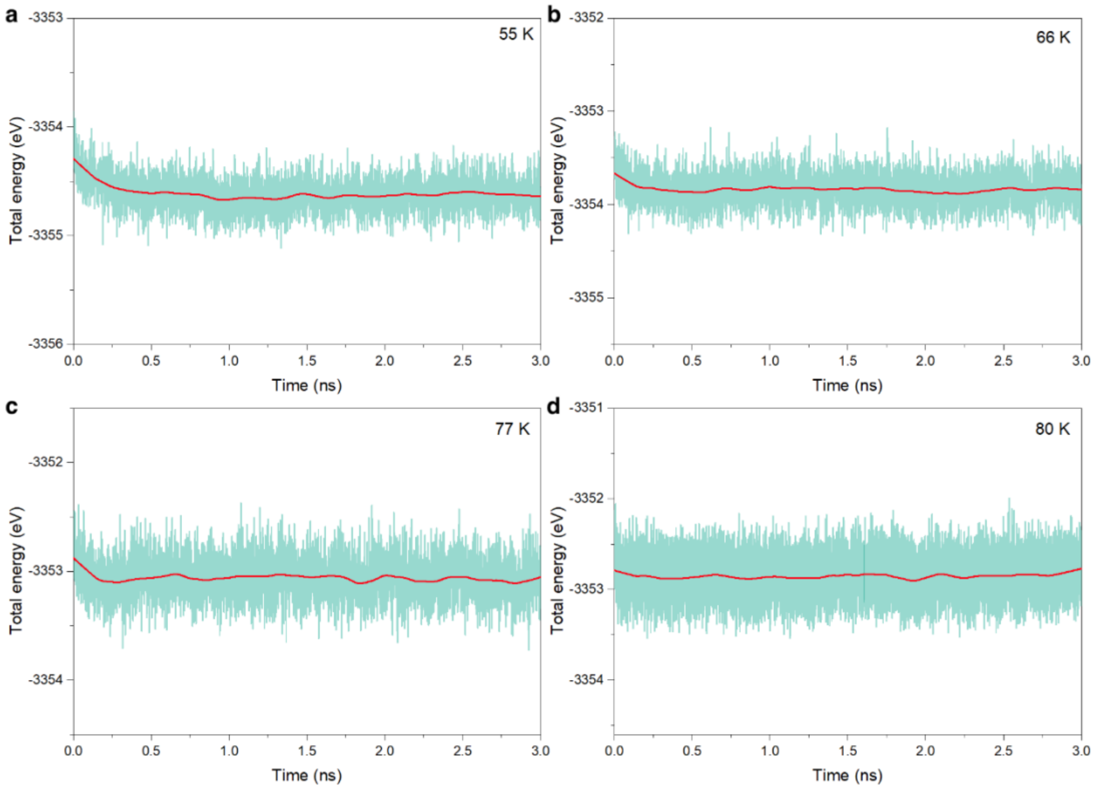

Supplementary Fig. S3 | Evolution of the energy over the MLP-MD trajectories for 24 H_2@Al-soc-MOF at (a-d) 55 K, 66 K, 77 K, and 80 K, respectively.

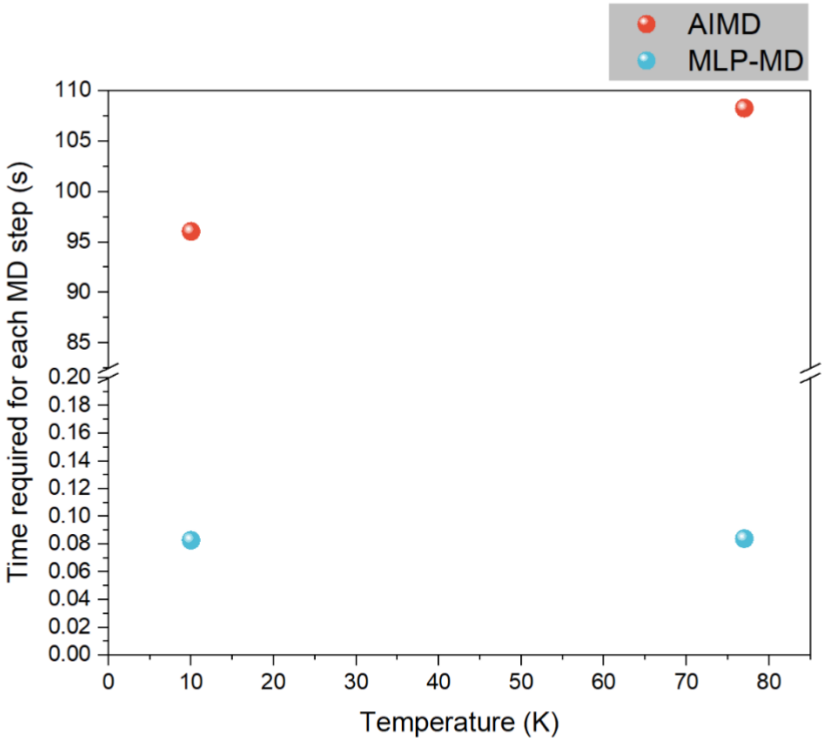

Supplementary Fig. S4 | The CPU time comparison between AIMD and MLP-MD simulations at 10K and 77K temperatures (24 H_2 loading per unit-cell). For each system, the same CPU (192-cores) was used to compute the average time per MD time step in seconds.

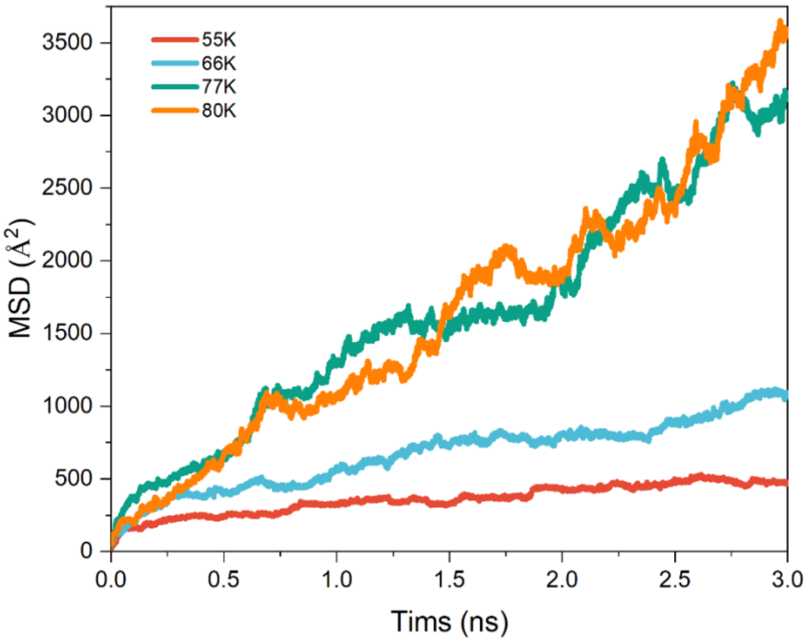

Supplementary Fig. S5 | MLP-MD simulated mean square displacement (MSD) of H_2 molecules (24 H_2 per unit-cell loading) *versus* simulation time at different temperatures.

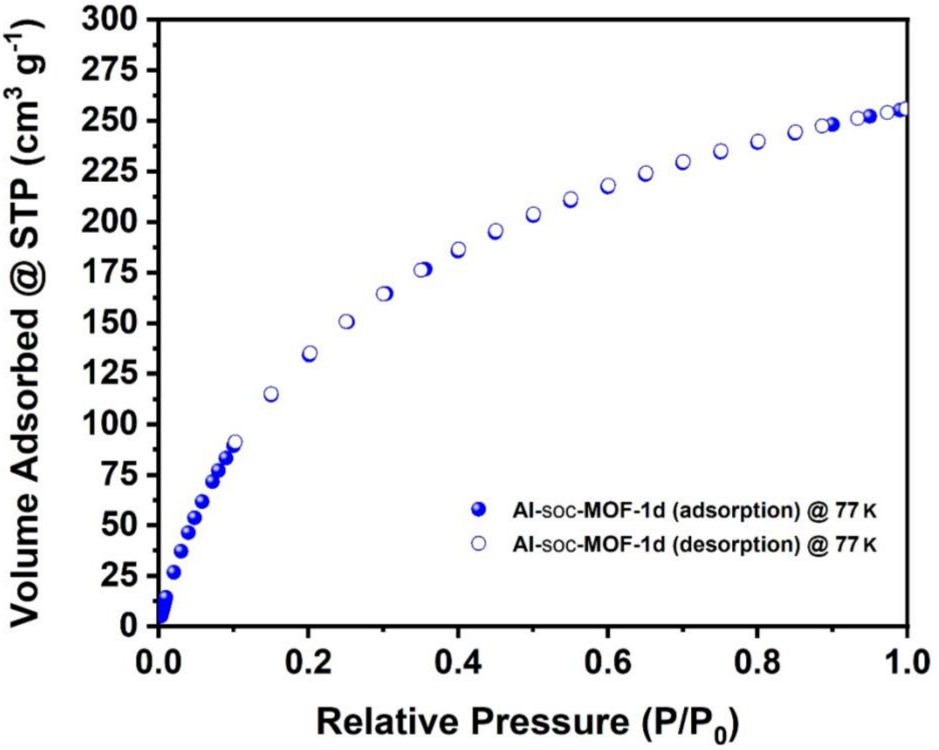

Supplementary Fig. S6 | Experimental H_2 adsorption isotherms collected at 77 K up to 1 bar.
